# Heatwaves and Mortality: The Influence of Choice of Definition and Lag

**DOI:** 10.1029/2025GH001675

**Published:** 2026-06-01

**Authors:** Yazan Alwadi, Fayez Abdulla, Yousef Khader, Wael K. Al‐Delaimy

**Affiliations:** ^1^ Environmental Health Department Harvard T.H. Chan School of Public Health Boston MA USA; ^2^ Civil Engineering Department Jordan University of Science and Technology Irbid Jordan; ^3^ Department of Public Health Jordan University of Science and Technology Irbid Jordan; ^4^ Herbert Wertheim School of Public Health and Human Longevity Science UC San Diego La Jolla CA USA

**Keywords:** heatwaves, all‐cause mortality, human mortality, public health, heatwaves and lagged effects, heatwave added effect

## Abstract

To investigate whether the reported additional effects of heatwaves on mortality are truly independent or instead reflect incomplete control for lagged temperature effects, and to assess whether this relationship varies by heatwave definition (e.g., minimum duration and intensity thresholds), as a potential explanation for heterogeneity in findings across the literature. We collected daily all‐cause mortality records spanning 19 years (2000–2018) in Amman, Jordan. Heatwaves were identified using 20 definitions with varying temperature thresholds (percentile‐based) and durations (2–5 consecutive days). Generalized Additive Models with penalized splines and Distributed Lag Non‐linear Models were used to assess temperature‐mortality associations and investigate how lag adjustment impacts the observed heatwave added effect. Without accounting for the lagged effects of temperature, heatwaves showed a significant additional effect on mortality in 7 out of 20 definitions. However, after properly controlling for these lagged effects, none of the heatwave definitions based on duration and threshold demonstrated a significant additional effect on mortality beyond what can be attributed to the cumulative (same day plus lagged) effect of daily temperature. The apparent added effect of heatwaves on mortality is largely explained by the cumulative impact of daily temperatures, suggesting that research and efforts might be more effective if focused on the cumulative effect of temperatures instead.

## Plain Language Summary

Heatwaves are often thought to cause additional deaths beyond those expected from hot weather. However, many studies have used different ways to define heatwaves and have not always accounted for the delayed effects of temperature over several days. We studied daily temperatures and deaths in Amman, Jordan, over 19 years to see whether heatwaves have an effect on mortality that is separate from the overall impact of high temperatures. We tested 20 different ways of defining heatwaves and compared models that either did or did not include delayed temperature effects. We found that when delayed temperature effects were not considered, heatwaves appeared to increase mortality. But when these delayed effects were properly included, the added effect of heatwaves disappeared. This suggests that the real driver of increased deaths is the cumulative effect of high temperatures, not heatwaves by themselves. Focusing public health efforts on protecting people from overall temperature extremes may therefore be more effective than concentrating only on periods defined as heatwaves.

## Key Points

- Heatwaves show no independent effect on all‐cause mortality once lagged temperature is considered
- Apparent added effects of heatwaves vanish after controlling for delayed temperature impacts
- The cumulative effect of daily temperatures, not heatwaves alone, drives mortality risk

## 1 Introduction

Heatwaves are extreme weather events defined by a set number of consecutive days with temperatures that exceed specific historic daily temperature thresholds within the same geographical location (Barnett et al., 2012; Perkins & Alexander, 2013). These events have been extensively studied due to their association with increased morbidity and mortality (Barnett et al., 2012; Campbell et al., 2018; Gasparrini & Armstrong, 2011; Ryti et al., 2016), increased energy consumption and water demand (Hatvani‐Kovacs et al., 2016), negative impacts on agriculture (Lobell et al., 2012), ecosystems and forests (Teuling et al., 2010), and natural and environmental hazards (Perkins et al., 2012). With the continuous increase of anthropogenic Green House Gases emissions, heatwaves events are expected to continue to grow in intensity and duration (Y. Sun et al., 2018; Ye et al., 2019; Zhang et al., 2020).

The wide range of effects associated with heatwaves, coupled with the observed and projected upward trends have drawn the attention of researchers and the volume of research dedicated to studying the effects of heatwaves on human health has increased in recent years (Campbell et al., 2018). However, despite this large volume of published research, the studies differ in their approach when defining what constitutes a heatwave (using different temperature thresholds and durations) as well as the methods used to demonstrate their effects through modeling (Ryti et al., 2016; Z. Xu et al., 2016).

To identify heatwaves effect on human health, studies have utilized temperature thresholds that range from the 90th percentile (Ahmadnezhad et al., 2013; Analitis et al., 2014; D’Ippoliti et al., 2010; Oudin Åström et al., 2015; Tong et al., 2014) to the 99th percentile (B. G. Anderson & Bell, 2009; Gasparrini & Armstrong, 2011; Hajat et al., 2006; Tong et al., 2014), and heatwave durations that ranged from 2 or more days (B. G. Anderson & Bell, 2009; Ma et al., 2015; Tong et al., 2014; Wang et al., 2012) or as high as 6 or more consecutive days (Ishigami et al., 2008). This wide range of duration and percentile to define any heatwave event led to varying or conflicting associations and effects.

Researchers have recognized that the inconsistency in heatwave definitions used across health studies is likely to introduce heterogeneity in the results, limit the transportability of findings, and make comparisons of health effects of heatwaves across studies more difficult (Z. Xu et al., 2016). This heterogeneity is bound to have contributed to some studies which have concluded that heatwaves have little to no added effect—defined as the effect above and beyond what would be expected from a hot days with comparable same‐day and lagged temperatures effects—beyond the risk of individual hot days when they are not classified as part of a heatwave (Barnett et al., 2012; Gasparrini & Armstrong, 2011; Guo et al., 2017), while others have concluded that there is a significantly higher and additional risk when several days consecutively come as a heatwave raising temperatures above a set threshold (B. G. Anderson & Bell, 2009; Hajat et al., 2006). To overcome this limitation, some studies reported the health effects of heatwaves by using various definitions of a heatwave within the same study (G. B. Anderson & Bell, 2011; Guo et al., 2017; Tong et al., 2014).

Another, potentially more influential, source of heterogeneity in the resulting health effects of heatwaves, which remains largely unexplored, is the variation in statistical methods used to quantify these effects. The lagged effect of temperature, in particular, has been ignored in some studies (Hutter et al., 2007; Lan et al., 2012; Tong et al., 2010), partially accounted for using 1 up to 6 lag days and moving averages (Hertel et al., 2009; Huang et al., 2010; Nitschke et al., 2011; X. Sun et al., 2014; Wang et al., 2012; Y. Xu et al., 2013) or fully accounted for using distributed Lag nonlinear models (DLNM) (Barnett et al., 2012; Gasparrini & Armstrong, 2011; Guo et al., 2017).

In this study, we analyzed data from Amman governorate, located in Jordan, spanning 19 years (2000–2018) to examine the association between heatwaves and all‐cause mortality across 20 different definitions of heatwaves and across 2 lag controls (DLNM and no lag) to determine the impact of the choice of definition and use of lag or not on these associations.

To our knowledge, this is the first study that explores the associations between heatwaves and health effects across different lag control scenarios and heatwaves definition.

## 2 Data and Methods

### 2.1 Data

**Figure 1:**
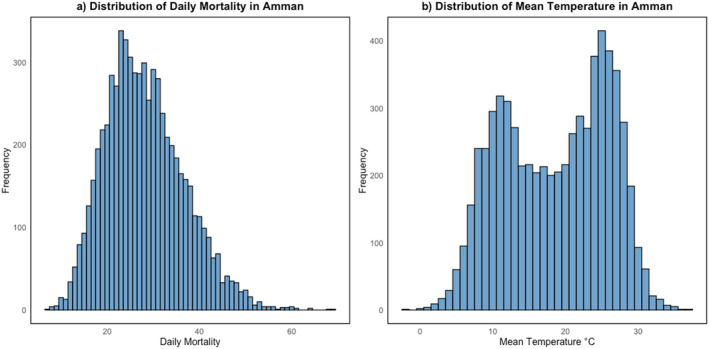
Distribution of all‐cause mortality and mean temperature in Amman 2000–2018.

We obtained an electronic copy in the form of a csv file of daily all‐cause mortality for Amman spanning from 1 January 2000 to 30 September 2018, sourced from the Jordanian Civil Status and Passport Department with a total of 184,167 deaths (Figure 1a). Concurrently, daily mean temperatures from Amman metrological station for the same period were collected from the Jordanian Ministry of Water and Irrigation (Figure 1b). Amman, covering an area of 7,579 km^2^, is a relatively small governorate known for its minimal temperature variation (Jaber, 2018). This spatial homogeneity in temperature is expected to mitigate errors associated with using a single station to represent the population's temperature exposure (Mistry et al., 2022).

### 2.2 Definitions of Heatwaves

To assess heatwaves associations with all‐cause mortality, we defined the presence of heatwaves using 20 definitions. Utilizing thresholds at the 90th, 92.5th, 95th, 97.5th, and 99th percentiles of year‐round mean temperatures during the 19 years of follow up, we identified heatwaves lasting a minimum of 2, 3, 4, and 5 consecutive days in duration. This approach yielded 20 unique heatwave definitions (Table 1). Heatwaves identification from the 19‐year data of temperatures for Amman was performed using the heatwaveR package (Schlegel & Smit, 2018), which provides tools to define and identify heatwave events based on various temperature thresholds and durations. This allowed for the systematic application of multiple heatwave definitions to the data set.

**Table 1:** All Heatwave Definitions Adopted in This Analysis Based on the Percentile Range for Average Daily Temperature and Using Different Temperatures as Thresholds for Defining an Extreme Heat Event (of Which Consecutively Happening for 2–5 or More Days It Is Defined as a Heatwave Event)

Days	Threshold				
	90th	92.5th	95th	97.5th	99th
2	Exceeds 90th for 2 or more days	Exceeds 92.5th for 2 or more days	Exceeds 95th for 2 or more days	Exceeds 97.5th for 2 or more days	Exceeds 99th for 2 or more days
3	Exceeds 90th for 3 or more days	Exceeds 92.5th for 3 or more days	Exceeds 95th for 3 or more days	Exceeds 97.5th for 3 or more days	Exceeds 99th for 3 or more days
4	Exceeds 90th for 4 or more days	Exceeds 92.5th for 4 or more days	Exceeds 95th for 4 or more days	Exceeds 97.5th for 4 or more days	Exceeds 99th for 4 or more days
5	Exceeds 90th for 5 or more days	Exceeds 92.5th for 5 or more days	Exceeds 95th for 5 or more days	Exceeds 97.5th for 5 or more days	Exceeds 99th for 5 or more days

## 3 Statistical Analysis

We began by examining the temporal patterns in heatwave frequency by calculating the number of heatwaves that exceeded the 95th percentile for 2 or more consecutive days each year from 2000 to 2018 in Amman. Following this, we assessed the crude univariate association between any particular heatwave definition and mortality by calculating the number of deaths observed during heatwaves identified using that heatwave definition, and then dividing this value by the number of heatwave days to calculate mean daily mortality.

To investigate the additional impact that heatwaves have beyond the effects of daily unlagged and lagged temperatures, we utilized a time series methodology. This involved fitting distinct negative binomial regression models to daily mortality counts for all days in the study period once for each heatwave definition.

### 3.1 Immediate Same Day Effect

To evaluate the immediate (unlagged) relationship between daily mean temperature and mortality we used penalized splines in a Generalized Additive Model (GAM) (Wood et al., 2016). We addressed time trends and seasonality with natural splines (4 degrees of freedom per year), controlled for day of the week categorically. We then added a binary categorical variable to identify heatwave days to capture the added effect of heatwaves.

### 3.2 Cumulative Effect

To evaluate the cumulative (immediate and delayed) effect of temperature we employed Distributed Lag Non‐Linear Model (DLNM). The DLNM framework enabled us to flexibly model non‐linear associations over time, accounting for lagged temperature effects (Gasparrini et al., 2010). For the DLNM model, we modeled mean temperature using a natural spline with two degrees of freedom positioned at the 50th and 90th percentiles for the exposure‐response dimension. We extended the lag to 10 days, employing a natural spline with two degrees of freedom evenly spaced on the log scale (Vicedo‐Cabrera et al., 2021). The DLNM parameter choices were informed by our previous works on Amman temperature—mortality association modeling (Alwadi et al., 2024). To account for time trends and seasonality, natural splines with 4 degrees of freedom per year were employed. Additionally, we controlled for the day of the week using categorical variables and introduced the same binary categorical indicator used in the immediate analysis to capture the added effect of heatwave.

We fitted both models 20 times to adjust for heatwaves identified using each of the 20 definitions independently. For each model fit we performed a separate timeseries analysis resulting in 40 distinct time series models (20 with lag and 20 without lag). This methodology enabled us to assess how the selection of definitions and the inclusion of lag controls influence the resulting associations.

### 3.3 Sensitivity Analysis

To assess the robustness of our findings and ensure that the results were not driven by specific data sources or modeling choices, we conducted a series of sensitivity analyses. First, we repeated the analysis using gridded temperature data from the ERA5‐Land data set (Copernicus Climate Change Service, 2024). Second, we varied the Distributed Lag Non‐Linear Model (DLNM) specifications used in the model.

All statistical analyses were performed using R statistical software version 4.1.2. The *dlnm* package (Gasparrini, 2011) was used for non‐linear delayed ambient temperature effects, which implements the Distributed Lag Non‐Linear Model framework and provides example code for applying the method. Plots were generated using ggplot2 package (Wickham et al., 2016), and GAM models were fitted using the mgcv package (Wood & Wood, 2015).

## 4 Results

### 4.1 Heatwave Detection

**Figure 2:**
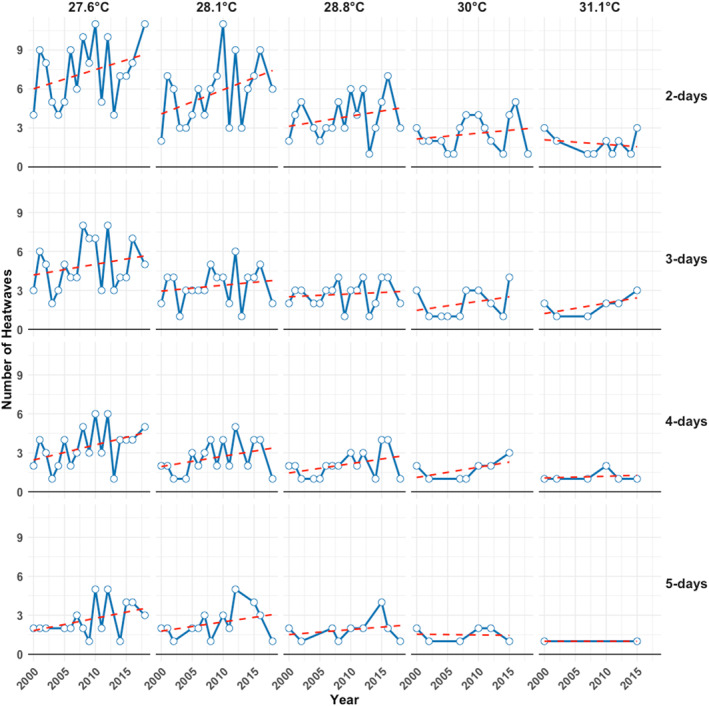
Temporal development of heatwaves identified by the 20 definitions in Amman (2000–2018).

The temporal change in heatwave frequency, based on 20 different definitions, is illustrated in Figure 2. A predominantly increasing pattern is observed across nearly all definitions. This suggests a general rise in heatwave occurrence over time, regardless of the specific definition used. Heatwaves identified using all the 40 different definitions are detailed in Table 2, including the minimum detection duration in days, the exceedance thresholds in both °C and %, the total number of days detected under each criterion, the count of unique heatwaves for each criterion, the average temperature of all heatwaves identified, the total observed mortality during these periods, and the average daily mortality for heatwaves detected using each criterion.

**Table 2:** Details of Heatwaves Detected Using 20 Different Definitions, Exceedance Percentiles, Minimum Durations, and Daily Mortalities

Min duration	Threshold percentile	Threshold °C	Total days	Number of heatwaves	Mean temperature °C	Total mortality	Daily mortality 95% CI
2	90	27.6	559	131	29.4	15,721	28.1 (27.7, 28.6)
2	92.5	28.1	409	102	29.9	11,715	28.6 (28.1, 29.2)
2	95	28.8	263	65	30.5	7,718	29.3 (28.7, 30.0)
2	97.5	30.0	131	38	31.6	3,893	29.7 (28.8, 30.7)
2	99	31.2	52	16	32.9	1,592	30.6 (29.1, 32.1)
3	90	27.6	473	88	29.6	13,367	28.3 (27.8, 28.7)
3	92.5	28.1	325	60	30.1	9,383	28.9 (28.3, 29.5)
3	95	28.8	225	46	30.7	6,629	29.5 (28.8, 30.2)
3	97.5	30.0	95	20	31.8	2,821	29.7 (28.6, 30.8)
3	99	31.2	42	11	33.0	1,296	30.9 (29.2, 32.5)
4	90	27.6	395	62	29.7	11,375	28.8 (28.3, 29.3)
4	92.5	28.1	271	42	30.2	7,949	29.3 (28.7, 30.0)
4	95	28.8	180	31	30.9	5,363	29.8 (29.0, 30.6)
4	97.5	30.0	71	12	32.1	2,146	30.2 (28.9, 31.5)
4	99	31.2	30	7	33.0	900	30.0 (28.0, 32.0)
5	90	27.6	307	40	29.9	9,093	29.6 (29.0, 30.2)
5	92.5	28.1	227	31	30.4	6,806	30.0 (29.3, 30.7)
5	95	28.8	124	17	31.2	3,862	31.1 (30.2, 31.1)
5	97.5	30.0	59	9	32.1	1,734	29.4 (28.0, 30.8)
5	99	31.2	10	2	32.7	310	31.0 (27.5, 34.5)

We found that the crude univariate daily mortality rates increased as the threshold and minimum duration of heatwave definitions became more stringent, with observed increases in daily mortality scaling with both the duration and severity of the heatwave threshold. For instance, using a minimum duration of 2 days, heatwaves detected at the 90th percentile threshold had a daily mortality rate of 28.1 (27.7, 28.6), while those detected at the 99th percentile had daily mortality rates of 30.6 (29.1, 32.1). This pattern persisted across varying minimum durations, with heatwaves exceeding the 95th percentile for 2, 3, 4, and 5 days showing daily mortality rates of 29.3 (28.7, 30.0), 29.5 (28.8, 30.2), 29.8 (29.0, 30.6), and 31.1 (30.2, 31.1), respectively (Table 2).

### 4.2 Daily Temperature Effect on Mortality

**Figure 3:**
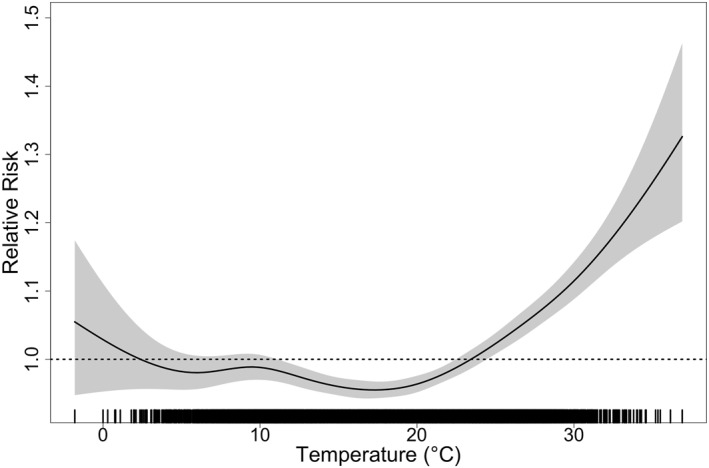
The unlagged (same day) mean temperature and all cause‐mortality associations resulting from the generalized additive model.

The immediate unlagged association between daily temperature and all‐cause mortality is illustrated in Figure 3, with a maximum heat‐attributable relative risk of 1.37 observed at a mean temperature of 37°C, and a maximum cold‐attributable relative risk of 1.1 at a mean temperature of −1.8°C.

**Figure 4:**
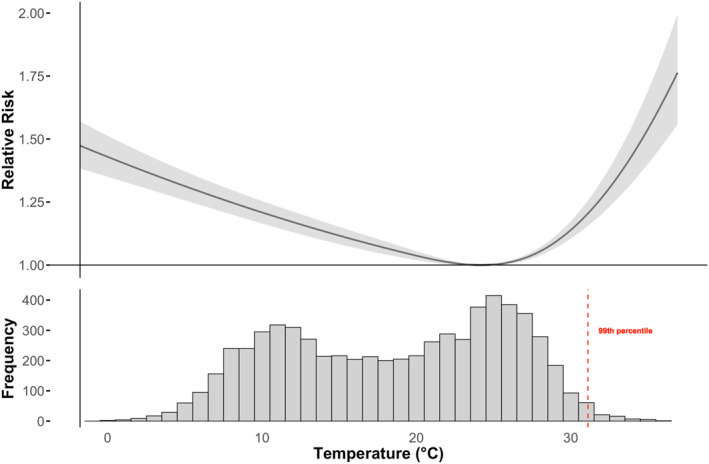
The cumulative lagged mean temperature and all cause‐mortality association with temperature distribution in Amman.

Amman's temperature distribution during the study period and its cumulative lagged association between temperature and all‐cause mortality is illustrated in Figure 4, with a maximum heat‐attributable relative risk of 1.76 observed at a mean temperature of 37°C and a maximum cold‐attributable relative risk of 1.43 at a mean temperature of −1.8°C.

### 4.3 Heatwave Effect on Mortality With Temperature Immediate Effect

**Figure 5:**
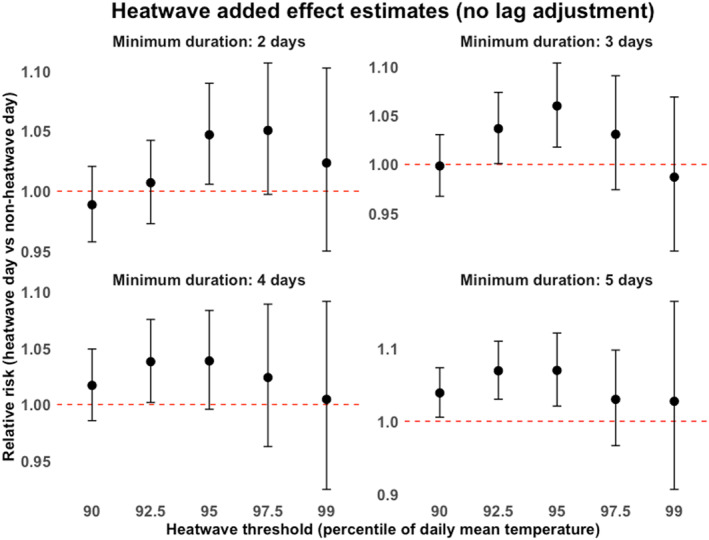
Added effects (95% CI) of heatwaves identified using minimum exceedance durations of 2, 3, 4, and 5 days with thresholds set at the 90th, 92.5th, 95th, 97.5th, and 99th percentiles in the unlagged temperature model.

The analysis capturing the added effect of heatwaves across all 20 definitions in the immediate unlagged temperature control model is shown in Figure 5. We found that 7 heatwave definitions had a significant added effect on all‐cause mortality in this model, specifically: daily mean temperatures that are higher than the 95th percentile for 2 or more days, higher than the 95th percentile for 3 or more days, higher than the 92.5th and 95th percentiles for 4 or more days, and higher than the 90th, 92.5th, and 95th percentiles for 5 or more days had significantly higher mortality rates compared to days with same temperatures that were not part of heatwaves.

### 4.4 Heatwave Effect on Mortality With Temperature Lagged Effect

**Figure 6:**
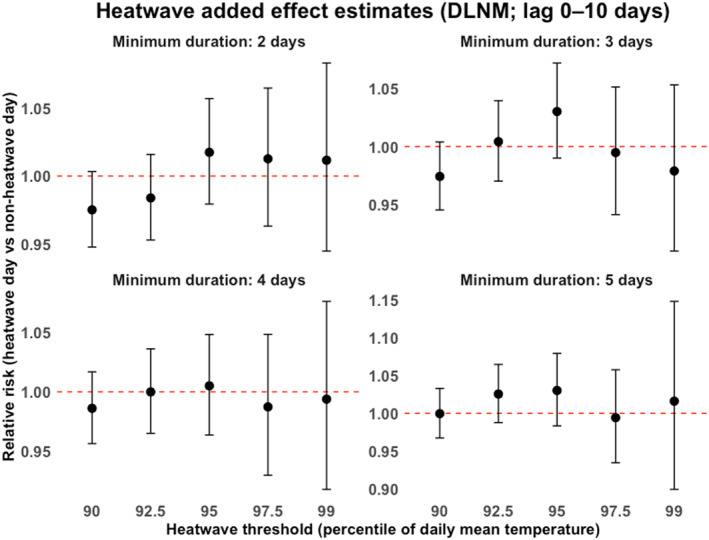
Added effects (95% CI) of heatwaves identified using minimum exceedance durations of 2, 3, 4, and 5 days with thresholds set at the 90th, 92.5th, 95th, 97.5th, and 99th percentiles in the cumulative (lagged) temperature model.

The added effect of heatwaves across all 20 definitions in the cumulative lagged temperature control model is illustrated in Figure 6. We found that all heatwaves lost significance when we adequately controlled for delayed effect of daily temperatures, regardless of the heatwave definition used.

### 4.5 Sensitivity Analysis Results

Repeating the analysis using temperature data from the ECMWF ERA5 reanalysis product instead of station‐based data from Amman yielded consistent results. Specifically, in models without lagged temperature adjustment, five heatwave definitions showed statistically significant added effects, whereas in the fully lagged DLNM models no significant added effects were observed for any heatwave definition (Figure S1 in Supporting Information S1).

We then repeated the analysis with additional adjustment for PM_2.5_ and relative humidity. As shown in Figure S2 in Supporting Information S1, these results were again consistent with the main findings: unlagged models identified significant added effects for six heatwave definitions, while the fully lagged DLNM models showed no significant heatwave‐related added effects across any definition.

## 5 Discussion

The observed rise in heatwaves' frequency in Amman aligns with findings from other studies (Perkins et al., 2012; Perkins‐Kirkpatrick & Lewis, 2020) and corresponds to regional temperature patterns, reflecting climate change and overall warming (Allen & Sheridan, 2016).

The crude univariate association observed between a stricter heatwave identification criterion and mean daily mortality (Table 2) is a relationship that is confounded by daily temperatures: Higher daily temperatures are associated with more extreme heatwaves (Domeisen et al., 2023; Perkins et al., 2012; Perkins‐Kirkpatrick & Lewis, 2020) and with increased daily mortality (Alahmad et al., 2020, 2023; Alwadi & Alahmad, 2024; Gasparrini et al., 2015, 2017). Therefore, adequately controlling for lagged temperature is essential to accurately isolating the added effect of heatwaves.

The association between daily temperatures and same day mortality has been extensively studied in the literature (Basu et al., 2008; Hutter et al., 2007; Lan et al., 2012; Tong et al., 2010). This also holds true for the delayed (lagged) effects of daily temperatures on mortality (Alahmad et al., 2020; Alwadi & Alahmad, 2024; Gasparrini et al., 2010, 2017; Vicedo‐Cabrera et al., 2021, 2021, 2021). We hypothesized that partial control for lagged effects of temperature can lead to residual confounding and bias the estimated added effects of heatwaves. Additionally, the variability in the observed effects of heatwaves in the literature may be partially attributable to heterogeneity in the control of lagged temperature effects, or the lack thereof.

We tested this hypothesis by estimating the added effect of heatwaves in both lagged and unlagged temperature control models using 20 different heatwave definitions for each. The unlagged models resulted in 7 out of 20 of the heatwave definitions having a significant added effect (Figure 5). However, upon controlling for lagged effects of temperature, all these added effects lost significance, regardless of the heatwave definition used (Figure 6). This observed loss of significance in the lagged models suggests that the initial significant results were potentially confounded by the lagged effects of temperature. These findings indicate that while researchers were attempting to reduce the observed heterogeneity in results by exploring and using multiple heatwave definitions, the bigger issue may have been the partial accounting for lagged effects of temperature.

The added effect of heatwaves and the lagged effect of temperature both describe the influence of previous days' temperatures on today's mortality. For heatwaves, these effects are considered only when several consecutive days meet specific criteria and are classified as a heatwave. In contrast, the lagged effect is calculated continuously, regardless of whether the days form a heatwave as the effect of the previous 10 days (in our analysis) on today's mortality is calculated for each day. This overlap in the calculated effects may explain the observed loss of significance in the added effect of heatwaves when the lagged effect is adequately controlled for.

Our results on heatwaves are consistent with findings from several studies in the literature. For example, one study that adequately controlled for the lagged effect of temperature examined the added effect of heatwaves using 12 heatwave definitions and data from 400 communities, and found no significant added effect of heatwaves (Guo et al., 2017). Similarly, another study that also adequately controlled for lagged effects of temperature, using data from 108 communities in the United States, found little to no added effect of heatwaves (Gasparrini & Armstrong, 2011). In contrast, many studies that reported a significant added effect of heatwaves did not adequately control for the lagged effects of temperature (B. G. Anderson & Bell, 2009; Hajat et al., 2006; Hutter et al., 2007; Lan et al., 2012; Nitschke et al., 2011; X. Sun et al., 2014; Tong et al., 2010; Y. Xu et al., 2013).

The non‐significant added effect of heatwaves does not negate their inherent dangers or the observed increase in mortality during these periods. On the contrary, our analysis demonstrates that mortality rates per day are notably higher during heatwaves, particularly with higher thresholds and longer durations (Table 2). However, these findings suggest that the increased mortality can be effectively explained by the lagged effects of daily temperatures without the need for explicitly controlling for heatwaves. This suggests that research, resources, and heat action plans might be more effective by focusing on the impacts of hot climatic conditions within the context of the cumulative effect of daily temperatures, rather than dividing efforts between cumulative effects and heatwaves.

While our study provides valuable insights into the relationship between heatwaves, daily temperatures, and mortality, several limitations should be acknowledged. First, the analysis focuses on all‐cause mortality, which may mask specific vulnerabilities in subpopulations or from particular causes of death. Second, the use of a single weather station to represent temperature exposure may not capture spatial variability across Amman. Additionally, while we controlled for lagged effects of temperature, other potential confounders, such as air pollution, humidity, and socio‐demographic factors, were not explicitly considered and could contribute to residual confounding. Lastly, our findings are based on data from Amman and may not be generalizable to other regions with different climate, socioeconomic, or population health characteristics.

## 6 Conclusion

Our study highlights the critical role of adequately controlling for lagged temperature effects when assessing the impact of heatwaves on mortality. While heatwaves are associated with higher daily mortality rates, the significant added effects observed in unlagged models appear to be confounded by these lagged effects. This overlap suggests that the observed increases in mortality are more consistent with the cumulative impact of daily temperatures, rather than heatwaves alone. Consequently, future research and public health strategies can benefit from focusing on understanding and addressing the cumulative effects of daily temperatures. An exclusive focus on heatwaves may overlook broader temperature‐related risks, and a more comprehensive approach could improve the interpretation of temperature–mortality relationships and inform more effective interventions for reducing heat‐related mortality.

## Ethics Statement

The authors provide the necessary ethical approval—IRB approval is obtained.

## Conflict of Interest

The authors declare no conflicts of interest relevant to this study.

## Supporting information

Supporting Information S1

## Data Availability

All temperature data used in this study were obtained from the Jordanian Ministry of Water and Irrigation. Mortality data were provided by the Jordanian Civil Status and Passport Department. Access to both data sets required official approvals and therefore cannot be directly shared by the authors. Interested researchers are encouraged to contact the Jordanian Ministry of Water and Irrigation (https://www.mwi.gov.jo/Default/En) for temperature data and the Jordanian Civil Status and Passport Department for mortality data.
