# Supplementary material for: Heatwaves and Mortality: The Influence of Choice of Definition and Lag
Source: Geohealth. 2026 Jun 1;10(6):e2025GH001675. doi: 10.1029/2025GH001675 (PMC13240302; doi:10.1029/2025GH001675)
Supplement: Supplementary file 1 — Supporting Information S1 [file GH2-10-e2025GH001675-s001.pdf]

GeoHealth

Supporting Information for

Heatwaves and Mortality: The Influence of Choice of Definition and Lag

Yazan Alwadi[1](Corresponding Author), Fayez Abdulla[2], Yousef Khader[3], Wael K Al-Delaimy[4]
Yazan_alwadi@fas.harvard.edu, fabdulla@just.edu.jo, yskhader@just.edu.jo, waldelaimy@ucsd.edu
[1] Environmental Health Department, Harvard T.H. Chan School of Public Health, 655 Huntington Ave, Boston, MA 02115, United States.
[2]Civil Engineering Department, Jordan University of Science and Technology, Irbid 22110, Jordan.
[3] Department of Public Health, Jordan University of Science and Technology, Irbid 22110, Jordan.
[4] Herbert Wertheim School of Public Health and Human Longevity Science, UC San Diego, 9500 Gilman Dr., La Jolla, CA 92093.

Contents of this file

Figures S1 to S2

Introduction

This Supporting Information provides additional results examining the added mortality effects of heatwaves under alternative heatwave definitions and model specifications. The figures present estimates derived from gridded daily mean temperature data obtained from the ERA5 reanalysis product. Heatwaves were defined using combinations of percentile-based temperature thresholds (90th to 99th percentiles) and minimum exceedance durations ranging from 2 to 5 consecutive days.

The analyses compare added heatwave effects estimated using two modeling approaches: (i) an immediate (unlagged) temperature model and (ii) a cumulative temperature model that fully accounts for lagged temperature effects. Figure S2 further extends this analysis by additionally controlling for ambient fine particulate matter ($PM_{2.5}$) concentrations and relative humidity (RH) to assess the robustness of the estimated heatwave effects to co-exposure adjustment.

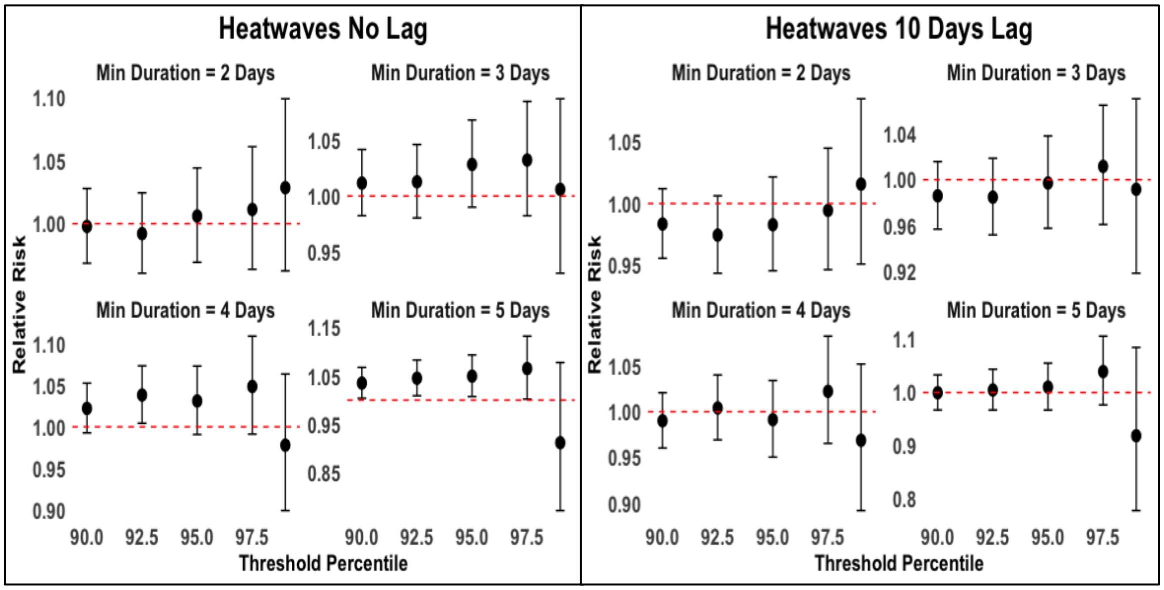

Figure S1. Added effects (95% CI) of heatwaves identified using minimum exceedance durations of 2, 3, 4, and 5 days with thresholds set at the 90th, 92.5th, 95th, 97.5th, and 99th percentiles in the cumulative (lagged) temperature model and immediate (unlagged model) using ERA5 grided temperature data.

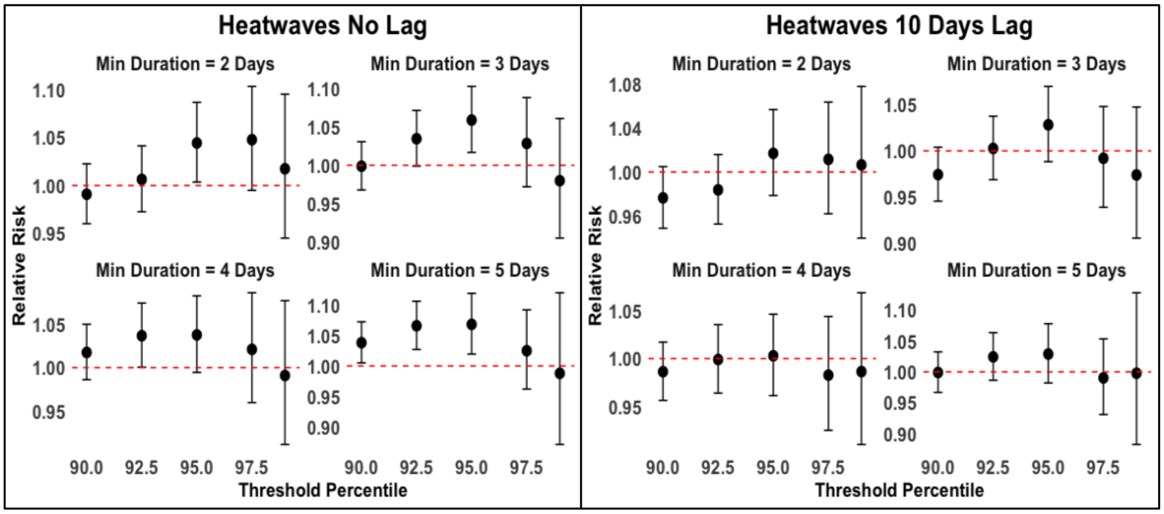

Figure S2. Added effects (95% CI) of heatwaves identified using minimum exceedance durations of 2, 3, 4, and 5 days with thresholds set at the 90th, 92.5th, 95th, 97.5th, and 99th percentiles in the cumulative (lagged) temperature model and immediate (unlagged model) and controlling for PM2.5 and RH.
